# Unusual worm-like radiopacities in the radiographs of patients with cervical spondylosis

**DOI:** 10.25122/jml-2022-0080

**Published:** 2022-11

**Authors:** Eric Chun-Pu Chu, Alan Te-Chang Chen, Ricky Chiang, Robert Trager

**Affiliations:** 1 New York Chiropractic and Physiotherapy Centre, EC Healthcare, Hong Kong SAR, China; 2 School of Health and Rehabilitation Sciences, The University of Queensland, Brisbane, Australia; 3 Connor Whole Health, University Hospitals Cleveland Medical Center, Cleveland, Ohio, United States of America

**Keywords:** cervical spondylosis, gold threads, radiographic artifacts, AP – anterior-posterior, NSAID – Non-steroidal anti-inflammatory drugs

## Abstract

This report describes three patients with cervical spondylosis whose diagnostic radiographs showed worm-like, irregularly curved radiopaque lines and strings in the head and neck region during routine chiropractic examinations. Such artifacts are frequently misinterpreted as parasitic infection, electrostatic discharges, detector image lag, fracture, or ligature wires. All three patients with worm-like radiopacities disclosed their 15–20 years of history of acupuncture treatment to relieve neck pain. The present cases of unexpected and coincidental findings may suggest a possible acupuncture-caused radiographic artifacts in the neck and jaw bones. In particular, the patient had previous gold thread treatments possibly associated with the observed radiographic artifacts. These cases may emphasize the importance of having a thorough understanding of patient history regarding unexpected radiographic artifacts.

## INTRODUCTION

Cervical spine radiographs are routinely performed to diagnose various abnormalities and pathologic conditions. Given the complex anatomy and projectional variances of the cervical spine, accurate interpretation of cervical radiographs can be challenging. For instance, a misdiagnosis for occipitalization can be caused by an abnormal head position at imaging [1]. When the head is too extended or flexed, superimposition of overlying anatomy in the upper cervical spine can be seen as pathologic conditions (*e.g*., dens fracture) [1]. Besides the imaging errors, other unexpected objects can cause additional difficulties in interpreting cervical spine radiographs. Although it is a general procedure that all metallic objects in the head and neck regions, including jewelry, removable dental appliances, and hair clips, are removed to avoid interference with the radiography, there have been few cases with imaging interferences by such metallic objects [2]. In this case report, we describe three cases that revealed numerous radiopaque and worm-like strings on the cervical radiographs during routine cervical radiographs performed in a chiropractic clinic to manage persistent neck pain [3]. Our observation may raise awareness of the potential radiographic interference caused by prior acupuncture treatments.

## CASE REPORT

The first patient was a 50-year-old Asian male who presented with a history of gradually worsening neck pain and radiating pain into the right upper extremity for 12 months. The patient walked in slouched and forward head postures. The patient also reported a chronic history of neck pain for 15 years, and he had tried pain killers, rehabilitation exercises, and different types of acupuncture procedures which provided minimum relief. After 20 sessions of chiropractic treatment over 2 months, the patient reported complete alleviation of symptoms. Consistently, the patient showed a reduced muscular hypertonicity, increased cervical range of motion, and resolution paresthesia.

The second patient was a 44-year-old Asian female who presented with neck pain without paresthesia for 15 years. The symptoms were often exacerbated during stress. Her symptoms were managed by medications and traditional Chinese medicine. She also disclosed that she received a special acupuncture technique using gold strings.

The third patient was a 65-year-old female who complained of chronic nuchal pain and bilateral upper arm pain for 20 years. She initially started with non-steroidal anti-inflammatory drugs (NSAID) treatment that failed to result in long-lasting improvement. The acupuncture and regular massage therapy offered her temporary relief.

**Figure 1:**
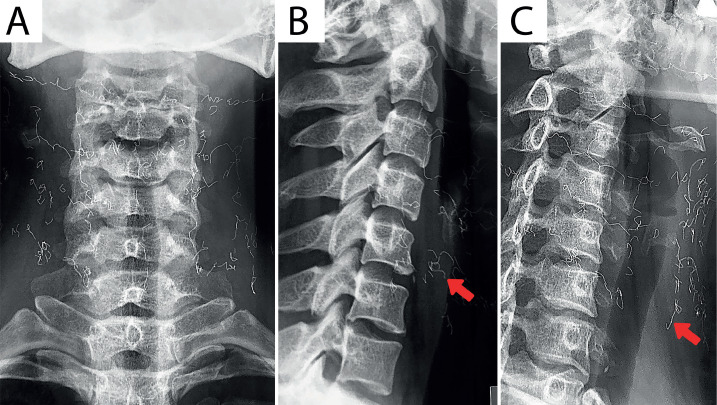
The cervical radiographs in A – AP; B – lateral; and C – oblique views indicate a loss of cervical lordosis, reduced intervertebral space between C4 and C5, osteophytes at C5-C7, and three layers of radiopaque strings over anterior and bilateral sides of the cervical spine.

*Investigation:* On physical examinations of the first patient, the chiropractor identified joint restrictions in the upper and mid cervical spine, limited cervical extension, and sensory deficit consistent with a C5 dermatomal distribution. The cervical radiographs in anterior-posterior (AP), lateral, and oblique views (Figure 1 A–C) indicated a loss of cervical lordosis, reduced intervertebral space between C4 and C5, osteophytes at C5-C7, and three layers of radiopaque strings over anterior and bilateral sides of the cervical spine.

**Figure 2:**
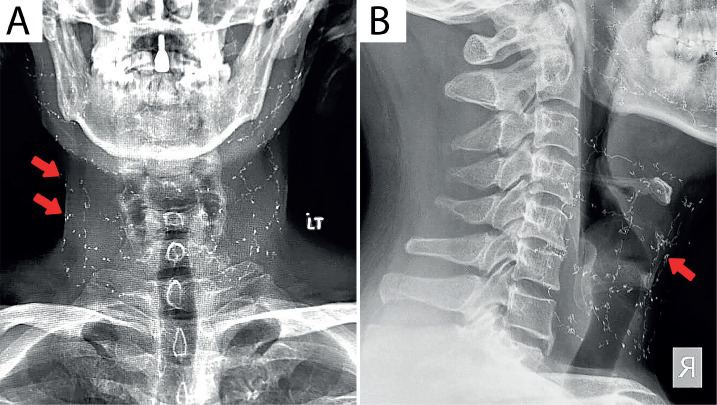
The cervical radiographs in A – AP; and B – lateral views indicate a loss of cervical lordosis, osteophytes at C5 and C6, and two layers of radiopaque strings over the anterior and bilateral sides of the cervical spine.

The second patient presented with joint restrictions in the mid-cervical region and limited cervical range of motion at right rotation and extension. The cervical radiographs in AP, lateral, and oblique views (Figure 2 A–B) indicated a loss of cervical lordosis, osteophytes at C5 and C6, and two layers of radiopaque strings over the anterior and bilateral sides of the cervical spine.

**Figure 3:**
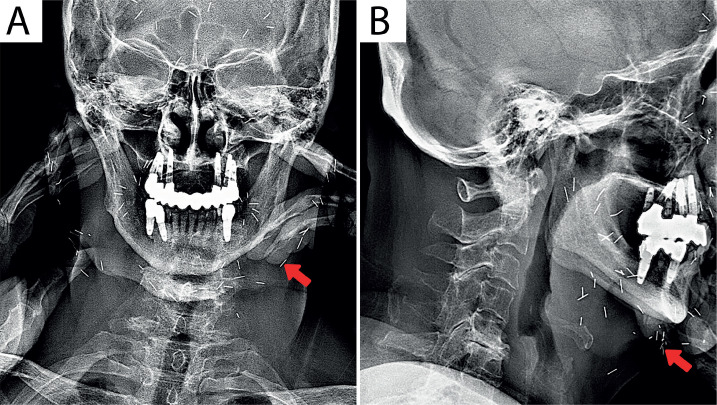
The cervical radiographs in A – AP; and B – lateral views indicate a loss of cervical lordosis, reduced intervertebral space between C4 and C5, osteophytes at C5-C7, and radiopaque string-like mesh over anterior and bilateral sides of the neck and molar area.

The cervical radiographs of patient 3 (Figure 3 A–B) indicated a loss of cervical lordosis, reduced intervertebral space between C4 and C5, osteophytes at C5-C7, and radiopaque string-like mesh over the anterior and bilateral sides of the neck and molar area.

*Diagnosis:* All patients were diagnosed with cervical spondylosis.

*Treatment:* We performed conservative intervention consisting of spinal manipulation of the restricted cervical segments, thermal ultrasound therapy and intermittent motorized traction of the cervicothoracic spine. The treatment regime is widely applied in chiropractic to relieve neck stiffness, restore normal joint motion, release intervertebral spaces, and decompress neural impaction. Treatment sessions were arranged three times weekly for two months.

*Outcome:* All patients reported that their symptoms progressively ameliorated from the second week onward and resolved completely within eight weeks. Neck mobility was mostly regained near the end of treatment. Following treatment, the patients reported neck pain reduced from 6-10/10 to 0/10 on the numeric pain rating scale. In addition, the neck disability index score declined from 32 - 66% to 0%. No adverse events occurred during the 3 months of care.

## DISCUSSION

Patients with cervical spondylosis are constantly reviewed by radiography of the cervical spine to examine the structures of bone, soft tissues and cartilage and their alignment [4]. To read, resolve, and make differential diagnoses without inaccuracies in patient imaging, clinicians need to be familiar with all possible radiographic artifacts [3]. Numerous metallic artifacts have been described in the literature, including bracelets, glasses, removable dentures, and piercings [2]. In some countries from South East Asia and Europe, unexpected metallic objects are inserted during musculoskeletal treatment and aesthetic procedures inside the body [5]. However, the radiological identification of such objects is rarely reported in the literature. Importantly, the unrecognized radiographic artifacts may obscure anatomical structures and critical pathologies, leading to misinterpretation of examination [6].

With the advancement of mobile phones, looking down on a mobile screen can lead to an overuse injury of the cervical spine resulting from the repetitive stress of prolonged forward head flexion [7]. Musculoskeletal pain is the leading cause of global disability, affecting poor physical activity, frailty, depression, and poor sleep quality. Besides well-established medical treatments, many patients with hardly curable musculoskeletal conditions seek alternative treatments available around the world. However, a relatively new treatment with limited clinical data is frequently associated with unexpected complications; thus, one should be cautious in consideration of such therapy [8]. In Korea, gold thread acupuncture is a common therapy for joint pain, wrinkles, and overall body wellness. The gold thread procedure is often perceived to induce “inner strength” of the body through insertions of small pieces of sterile gold thread by acupuncture needles [9]. However, a previous report showed that gold threads used for pain management of rheumatoid arthritis were left around the deformed hands and feet in the body for years [5]. In another report, gold threads gradually migrated and fragmented over time, damaging the surrounding tissue [6, 10, 11]. For example, the gold fragments injected on the back may travel through the blood vessels to the lower extremity, causing secondary inflammatory reactions which require systemic steroids and antibiotics [12].

Gold threads consist of a 24-carat-gold surgical suture implanted into the skin with a needle in a mesh or fan-like pattern. In theory, the gold thread mesh was aimed to build new collagen around the suture by stimulating the surrounding tissue matrix [5, 13]. It is believed that the denser threads in the “mesh” or “fan” lead to a tighter lift. Given the highly vascularized tissue capsule surrounding the gold threads, gold thread implantation is believed to promote angiogenesis, followed by further induction of mast cell growth over time [9, 14].

However, numerous previous reports suggested that such metallic threads may obstruct radiographic assessment by entangling critical anatomical structures [13]. In addition, fragmented gold threads can significantly interfere with the radiographic analysis. Besides the above-mentioned strings, other artifacts are often observed on radiographs, such as motion artifacts, image compositing, and grid cut-off, consequently complicating the process of obtaining a good radiographic interpretation. Thus, a thorough understanding of all possible types of artifacts is primarily required for cervical radiography [6]. However, the study has limitations that include a small sample size or lack of a control group.

## CONCLUSION

There are various causes for artifacts appearing on skeletal radiographs. Artifacts may obscure anatomical structures and/or mask critical structures/pathologies that may result in misinterpretation and inaccurate diagnosis. Careful and detailed patient history should be reviewed, particularly with acupuncture such as gold thread treatments.

## Data Availability

Further data is available from the corresponding author upon reasonable request.
